# Down-regulation of cylindromatosis protein phosphorylation by BTK inhibitor promotes apoptosis of non-GCB-diffuse large B-cell lymphoma

**DOI:** 10.1186/s12935-021-01891-2

**Published:** 2021-04-07

**Authors:** Xin Xu, Ting Wei, Weijie Zhong, Rosalind Ang, Ye Lei, Hui Zhang, Qingshan Li

**Affiliations:** 1 The First Affiliated Hospital, Jinan University, Guangzhou, Guangdong 510630 People’s Republic of China; 2 Guangzhou First People’s Hospital, School of Medicine, South China University of Technology, Guangzhou, Guangdong 511458 People’s Republic of China; 3 Department of Hematology, Guangzhou First People’s Hospital, School of Medicine, South China University of Technology, Guangzhou, Guangdong 510180 People’s Republic of China; 4 Department of Geriatrics, Hematology and Oncology Ward, Guangzhou First People’s Hospital, School of Medicine, South China University of Technology, Guangzhou, Guangdong 510180 People’s Republic of China; 5 Precision Immunology Institute, Mount Sinai School of Medicine, New York, NY 10029 USA; 6 Institute of Immunology and Molecular Medicine, Jining Medical University, Jinan, Shandong 272067 People’s Republic of China; 7 Department of Hematology, Guangzhou Red Cross Hospital, Jinan University, No. 396 Tongfuzhong Road, Haizhu District, 510220 Guangzhou, Guangdong People’s Republic of China

**Keywords:** Diffuse large B-cell lymphoma, Cylindromatosis, Phosphorylation, Bruton’s tyrosine kinase inhibitor, Apoptosis, Rituximab, Resistance

## Abstract

**Background:**

Non-germinal center B-cell-like diffuse large B-cell lymphoma (non-GCB-DLBCL) has worse clinical outcome than GCB-DLBCL, and some relapsed/refractory non-GCB-DLBCL (R/R non-GCB-DLBCL) are even resistant to CD20 monoclonal antibody (rituximab). Bruton’s tyrosine kinase inhibitors (BTKis) are new drugs for B-cell lymphoma. BTKis can promote apoptosis of DLBCL by inactivating nuclear transcription factor κB (NFκB) signaling pathway. Cylindromatosis (CYLD) is a tumor suppressor and ubiquitinase. CYLD can inactivate NFκB signaling pathway through ubiquitination and regulate the apoptosis of hematological tumors. The ubiquitination of CYLD can be regulated by phosphorylation, suggesting that the regulation of CYLD phosphorylation can be a potential mechanism to promote the apoptosis of hematological tumors. Therefore, we hypothesized that BTKis could promote the apoptosis of non-GCB-DLBCL by regulating the phosphorylation of CYLD, especially in rituximab resistant cases, and we proved this hypothesis through both in vivo and in vitro experiments.

**Methods:**

The baseline expression levels of CYLD phosphorylation in non-GCB-DLBCL patients and cell lines were detected by Western Blotting. The non-GCB-DLBCL cell lines were treated with BTKis, and apoptosis induced by BTKis treatment was detected by Western blotting, cell viability assay and Annexin V assay. To verify whether the effect of BTKis on apoptosis in non-GCN-DLBCL cells is CYLD dependent, the expression of CYLD was knocked down by lentiviral shRNAs. To verify the effect of BTKis on the phosphorylation of CYLD and the apoptosis in vivo and in rituximab resistant non-GCB-DLBCL, the xeograft model and rituximab resistant non-GCB-DLBCL cells were generated by tumor cell inoculation and escalation of drug concentrations, respectively.

**Results:**

BTKis induced apoptosis by down-regulating CYLD phosphorylationin in non GCB-DLBCL, xenograft mouse model, and rituximab-resistant cells, and this effect could be enhanced by rituximab. Knocking-down CYLD reversed apoptosis which was induced by BTKis. BTKis induced CYLD-dependent apoptosis in non-GCB-DLBCL including in rituximab-resistant cells.

**Conclusions:**

The present results indicated that CYLD phosphorylation is a potential clinical therapeutic target for non-GCB-DLBCL, especially for rituximab-resistant relapsed/refractory cases.

## Background

Diffuse large B-cell lymphoma (DLBCL) is the most common invasive non Hodgkin’s lymphoma (NHL), which can be divided into two categories: germinal center B-cell-like DLBCL (GCB-DLBCL) and non-germinal center B-cell-like DLBCL (non-GCB-DLBCL) [1]. Although the combination of CD20 monoclonal antibody (rituximab, RTX) and CHOP (cyclophosphamide, vincristine, adriamycin, and prednisone) (RCHOP) has greatly improved the therapeutic effect of DLBCL [2], the therapeutic effect and overall prognosis of non-GCB-DLBCL are still worse than that of GCB-DLBCL [3]. In non-GCB-DLBCL, the overall recurrence rate after treatment is more than 30%. Some refractory/relapsed cases are resistant to rituximab, which makes the treatment even more difficult [4, 5]. It is urgent to develop new drugs to improve the efficacy of non-GCB-DLBCL, especially for rituximab resistant non-GCB-DLBCL [6–9].

BTK inhibitors (BTKis) ibrutinib and acalabrutinib are important new drugs for the treatment of B-cell lymphoma including DLBCL in recent 10 years [10]. Ibrutinib was the first covalent BTK inhibitor [11]. Ibrutinib compound PCI-32765 inhibited BTK phosphorylation which blocks B-cell activation and is efficacious in models of autoimmune disease and B-cell malignancy [12]. Ibrutinib has been used as the first-line therapy for chronic lymphocytic leukemia/small lymphocytic lymphoma (CLL/SLL) and the second-line therapy for mantle cell lymphoma (MCL) since the publication of the 2016 National Comprehensive Cancer Network (NCCN) guidelines (version 3) [13, 14]. In DLBCL, Phase II clinical trials in patients with refractory DLBCL found that ibrutinib was more effective against non-GCB-DLBCL compared with GCB-DLBCL. The possible mechanism was that non-GCB-DLBCL had continuous activation of the B-cell receptor (BCR) signaling pathway than GCB-DLBCL, which could be inhibited by ibrutinib switch more tumor cells transfer from survival to death [15, 16]. Active BCR signaling activates multiple downstream pathways [17], one is NFκB. NFκB activation plays crucial role in tumor development and progression [18]. BTKis has been shown to inhibit NFκB activation in hematological malignancies [19]. But in non-GCB-DLBCL, especially in rituximab-resistant non-GCB-DLBCL, whether BTKis could improve apoptosis through down-regulating NFκB activation and the underlying mechanism remain unclear.

CYLD was first identified in familial cylindroma and was shown to be a tumor suppressor [20]. CYLD had ubiquitination function and previous studies have found that CYLD can inactivate NFκB signaling pathway through ubiquitination and regulate the apoptosis of hematological tumors [21]. There are several regulatory mechanisms of CYLD function. CYLD gene mutation can regulate the growth of leukemia cells by down regulating mitotic kinase 1 and blocking nuclear translocation in B-lymphocytic leukemia and in DLBCL. Mucosa associated lymphoma translocation gene 1(MALT1) gene can cause the cleavage of CYLD and promote the proliferation of tumor [21]. Besides gene mutation and protein cleavage, phosphorylation is one of the most important ways to regulate the activity of CYLD. Previous literature reported that serine in 418–444 region of CYLD was the main site of phosphorylation. When serine in this region is mutated into alanine, CYLD cannot be phosphorylated, and its activity will be significantly enhanced, which will impact on CYLD ubiquitination function, resulting in the generation of apoptosis signal through NFκB signaling pathway [22, 23]. Our previous research has demonstrated that adult T-cell leukemia/lymphoma (ATLL) had high level of CYLD phosphorylation, down-regulating CYLD phosphorylation could increase CYLD deubiquitinase activity and then promoted apoptosis in ATLL [24]. Whether CYLD phosphorylation is also a therapeutic target for non-GCB-DLBCL is unknown. Previous research suggested that BTKis ibrutinib can largely increase CYLD miRNA transcription, which means increasing CYLD activity, could inhibit cells proliferation in CLL [25]. But whether BTKis can regulate CYLD phosphorylation and to improve cell apoptosis in hematopoietic malignancies, especially in non-GCB-DLBCL, is also unknown.

In this study, we aimed to demonstrate that BTKis could induce apoptosis by inhibiting CYLD phosphorylation in non-GCB-DLBCL, including in rituximab-resistant non-GCB-DLBCL. The results will further our understanding of the molecular mechanism of apoptosis in non-GCB-DLBCL cells and provide a molecular basis for pharmacologic potential target in non-GCB-DLBCL, especially in the rituximab-resistant R/R non-GCB-DLBCL cases.

## Materials and methods

### Antibodies and reagents

Antibodies were purchased from the indicated suppliers: glyceraldehyde3-phosphate dehydrogenase (GAPDH; clone D-6; Santa Cruz Biotechnology, Santa Cruz, CA, USA). phosphorylated-CYLD (p-CYLD) (Ser418; #4500), CYLD (D1A10; #8462), phosphorylated-BTK (p-BTK) (Tyr223; #5082), BTK (D3H5; #8547), cleaved caspase-3 (Asp175; #9661), and β-actin (8H10D10; #3700) (all Cell Signaling Technology, Danvers, MA, USA). Ibrutinib (PCI-32765) and acalabrutinib (ACP-196) (Selleck Chemicals, Houston, TX, USA). CD20 monoclonal antibody (rituximab) (Novartis, Basel, Switzerland). DMSO (as control) (Selleck Chemicals, Houston, TX, USA).

### Human lymphoma samples

Five tumor invasive lymph node samples from newly diagnosed non-GCB-DLBCL lymphomas and five inflammatory hyperplasia lymph node samples from lymphadenitis patients as control were collected at Guangzhou First People’s Hospital between 2017 and 2019, according to protocols approved by Guangzhou First People’s Hospital in 2017 (K-2017-105-01). Non-GCB-DLBCL were classified according to the WHO 2016 criteria, based on morphologic features and immunophenotype [26]. Lymphoma and lymphadenitis patients were defined by experienced pathologists. All samples were obtained from five pairs of lymphoma and lymphadenitis patients, at different time, and all samples were frozen in optimum cutting temperature compound (OCT). We did protein extration immediately when we got the samples and run for Western Blotting. Protein extration was followed method for membrane or structural protein extraction according to reference [27].

### Cell lines and cell culture

The Jurkat T-cell 3T8 and the human peripheral blood B lymphocyte cell line RPMI 1788 were selected as the negative controls because of these cell lines had low basal levels of CYLD phosphorylation [28]. And the non-GCB-DLBCL cell lines OCI-Ly10 and HBL-1 DLBCL cell lines were selected to carry out experiments of this study. All the above cell lines were purchased from ATCC (Shanghai, China) with authentication at 2017 and cultured in RPMI 1640 medium (Hyclone) containing 10% fetal bovine serum (Hyclone), 4 mM l-glutamine (Sigma-Aldrich, St. Louis, MO, USA), 100 U/ml penicillin (Hyclone, Logan, UT, USA), and 100 U/ml streptomycin (Hyclone, Logan, UT, USA). All cells were cultured in a humidified chamber at 37 °C with an atmosphere of 5% CO_2_. All cell lines had been tested for mycoplasma contamination. CD20 monoclonal antibody (rituximab)—sensitive (Ly10-S) and resistant (Ly10-R) OCI-Ly10 cells were conducted in this study. In order to construct Ly10-R, parental OCI-Ly10 cells were cultured at 37°C and 5% CO_2_. Once they reached logarithmic growth phase, they were exposed to gradually increasing concentrations of rituximab (0, 16, 32, 64, 128 μg/ml) and supplemented with human serum (1:1000–1:1.875). After 24 h exposure to rituximab at each concentration, the cells were centrifuged, and the medium was replaced with fresh RPMI 1640. Cells were then allowed to regrow for a minimum of 3 days, and once exponential log phase of growth was reached, the procedure was repeated for a total of 10 passages at which time functional assays (i.e., complement-dependent cytotoxicity, CDC) showed maximal inhibition of rituximab-associated biological activity [29].

### Mouse xenograft model

All animal studies were carried out according to the Institutional Animal Care and Use Committee protocols approved by Guangzhou First People’s Hospital in 2017 (K-2017-105-01) for animal welfare. BALB/c-nu mice (Guangdong Laboratory Animal Center, China) were inoculated subcutaneously with 5.0 × 10^6^ OCI-Ly10 cells in a suspension containing Matrigel (Corning, New York, USA) [30]. When the implanted tumors reached ~ 100 mm^3^ the mice were randomly assigned to two groups. One was treated once daily with ibrutinib (PCI-32765; 12 mg/kg; methylcellulose coating) by oral and another was treated by methylcellulose as control (n = 10 mice per group). The tumors were measured twice a week and tumor volume was calculated as V = (length × width^2^)/2 [31].

### Plasmids and transfection

A non-targeting shRNA or CYLD-targeting shRNA (SHCLNG-NM_015247, TRCN00000 39629) encoded by the lentiviral vector pLKO.1-puro were obtained from Sigma-Aldrich (St. Louis, MO, USA). Retroviral transduction of non-GCB-DLBCL cells was performed as previously described [32]. Lentivirus particles encoding shRNA were pseudotyped and packaged in a similar manner as retroviruses. Non-GCB-DLBCL cells were stably selected using puromycin after 48 h transduction.

### Western blotting

For Western Blotting, 1 × 10^6^ cells in each sample were lysed in buffer containing 1% Triton X-100 as previously described [33]. For each sample, 50 µg of protein was resolved by 10% SDS-PAGE and transferred to nitrocellulose membranes. Protein detection was carried out by incubating membranes with primary antibodies overnight at 4 °C, followed by incubating membranes with secondary antibody (according to different primary antibodies) for 1 h at room temperature. Blotting visualization was carried out using chemiluminescence (ECL reagent, Thermo Fisher, Waltham, MA, USA). Western Blotting analysis of lysates from cell lines were performed three times.

### ATP viability assay

Cells were seeded 2.5 × 10^4^ cells/well in 96-well plates. Viability was analyzed using the CellTiter-Glo^®^ Luminescent Cell Viability Assay kit (Promega, Madison, WHCA, USA) according to the manufacturer's instruction.

### NFκB activity assay

NFκB activities were measured by NFκB (p65) Transcription Factor Assay Kit (Cayman Chemical, Ann Arbor, MI, USA) [34]. Cells were lysed and the nuclear fractions were extracted, then the protein content were assayed by the Bradford method [35]. Each microplate well pre-coated with the dsDNA which was capable to bind with phosphorylated p65 subunit of the NFκB, and qual amounts of 20 µg protein were loaded into each well. Anti-phosphorylated p65 primary antibody, HRP-conjugated secondary antibody, and chromogen substrate solutions were added sequentially. The light absorbance of the wells was read at 450 nm and reported as the estimate of the NFκB activities.

### Apoptosis assay

The apoptotic cell population was quantified using an ApoDETECT Annexin V-FITC Kit (Life Technologies, Carlsbad, CA, USA) according to the manufacturer’s procedure. Briefly, cells were washed with ice-cold PBS and re-suspended in 1 × binding buffer at a concentration of 5 × 10^6^ cells/ml. Annexin V-FITC (10 ml) was added to 190 ml of cell suspension and incubated at room temperature for 10 min. After washing with 1 × binding buffer, the cells were re-suspended in 190 ml of binding buffer with 10 ml of 20 mg/ml propidium iodide and analyzed by flow cytometry (FACS Canto II; BD Bioscience).

### Complement-dependent cytotoxicity (CDC) by ^51^Cr-release assay

To show a decrease in biological activity in the CD20 monoclonal antibody (rituximab)—resistant (Ly10-R) OCI-Ly10 cell line, standard ^51^Cr release assays were done to asses rituximab-mediated CDC^34^. CD20 monoclonal antibody (rituximab)—sensitive (Ly10-S) or resistant (Ly10-R) OCI-Ly10 cells were labeled for 2 h at 37 °C with 3.7 MBq of 51Cr (100 uCi). The radioactive excess was washed out thrice in PBS and the tumor cells were re-suspended on RPMI-10 medium. 100 μL aliquots (adjusted cell density to 1 × 10^5^ cells per well) were placed in 96-well plates. Subsequently, Ly10-S or Ly10-R cells were pre-incubated with RPMI-10, rituximab (0, 16, 32, 64, 128 μg/ml) in combination with human serum (dilution 1:4). Pooled human serum collected from healthy donors was used as a source of complement. Serum samples were obtained under protocol approved by Guangzhou First People’s Hospital in 2017 (K-2017-105-01). Subsequently, cells were incubated at 37 °C, 5% CO_2_ for 6 h. Finally, the 96-well plates were centrifuged at 1400 rpm (300 *g*), 4 °C for 5 min and the supernatant of each well was collected individually and γ ray emission was measured by the Packard Auto-Gamma Cobra II series counting system (IBM, Inc.). All samples were run in triplicate in three different sets of experiments. Results are reported as a mean values with standard deviation (SD).

### Complement-dependent cytotoxicity (CDC) by cell counting kit-8 (CCK-8) assay

To show biological activity in the CD20 mAb (rituximab)—resistant (Ly10-R) OCI-Ly10 cell line, standard Cell Counting Kit-8 (CCK-8) assay were done to asses rituximab-mediated CDC. CD20 monoclonal antibody (rituximab)—sensitive (Ly10-S) or resistant (Ly10-R) OCI-Ly10 cells were centrifuged at 1200 rpm for 5 min, and then mixed with RPMI-10 in combination with human serum. Cell density was adjusted to 5 × 10^3^ cells per well and inoculated in 96 well cell culture plate with rituximab. Rituximab was added according to the above different concentrations (0, 16, 32, 64, 128 μg/ml), and three parallel microplate wells were set for each concentration. 10 µl CCK-8 reagent was added into each well at 37 °C and 5% CO_2_ incubator for 2 h respectively, and the OD value of each well at 450 nm was measured by enzyme-linked immunosorbent assay after 4 h maintenance incubation. The inhibition rate was calculated by the following formula:growth inhibition rate = [OD value of the control g − roupOD value of the experimental group)/(OD value of the control group − OD value of the blank group) × 100%. All samples were run in triplicate in three different sets of experiments. Results are reported as a mean values with standard deviation (SD).

### Flow cytometry

In order to construct rituximab resistant OCI-Ly10 cells (OCI-Ly10R), parental OCI-Ly10 cells were were exposed to sequentially increasing concentrations of rituximab (0, 16, 32, 64, 128 μg/ml). The CD20 expression levels on rituximab-sensitive (OCI-Ly10S) or resistant OCI-Ly10 cells (OCI-Ly10R) were detected by flow cytometry, respectively. Cell density was adjusted to 2 × 10^6^/ml. Surface CD20 monoclonal antibody (mAb) (Dako, Carpinteria, CA, USA) was labeled with fluorescein isothiocyanate (FITC), and Mouse Ig labeled with FITC (Coulter Corporation, Hialeah, FL, USA) was used for negative control. Cells were stained with anti-CD20-FITC antibody for 1 h at room temperature and then analyzed with flow cytometer (FACS Canto II; BD Bioscience). Datas were analyzed with FlowJo 10 software.

### Statistical analysis

All analyses were performed using SPSS 17.0 software. Numerical data are presented as the means ± standard deviation (mean ± SD). The difference between two groups was determined using Two-tailed independent-sample Student’s *t’/t* tests. Multivariate analysis of variance (two-way ANOVA) was used for comparisons among multiple groups. A P-value of 0.05 was considered to indicate a statistically significant difference.

## Results

### Phosphorylated CYLD elevated in non-GCB DLBCL

**Fig. 1:**
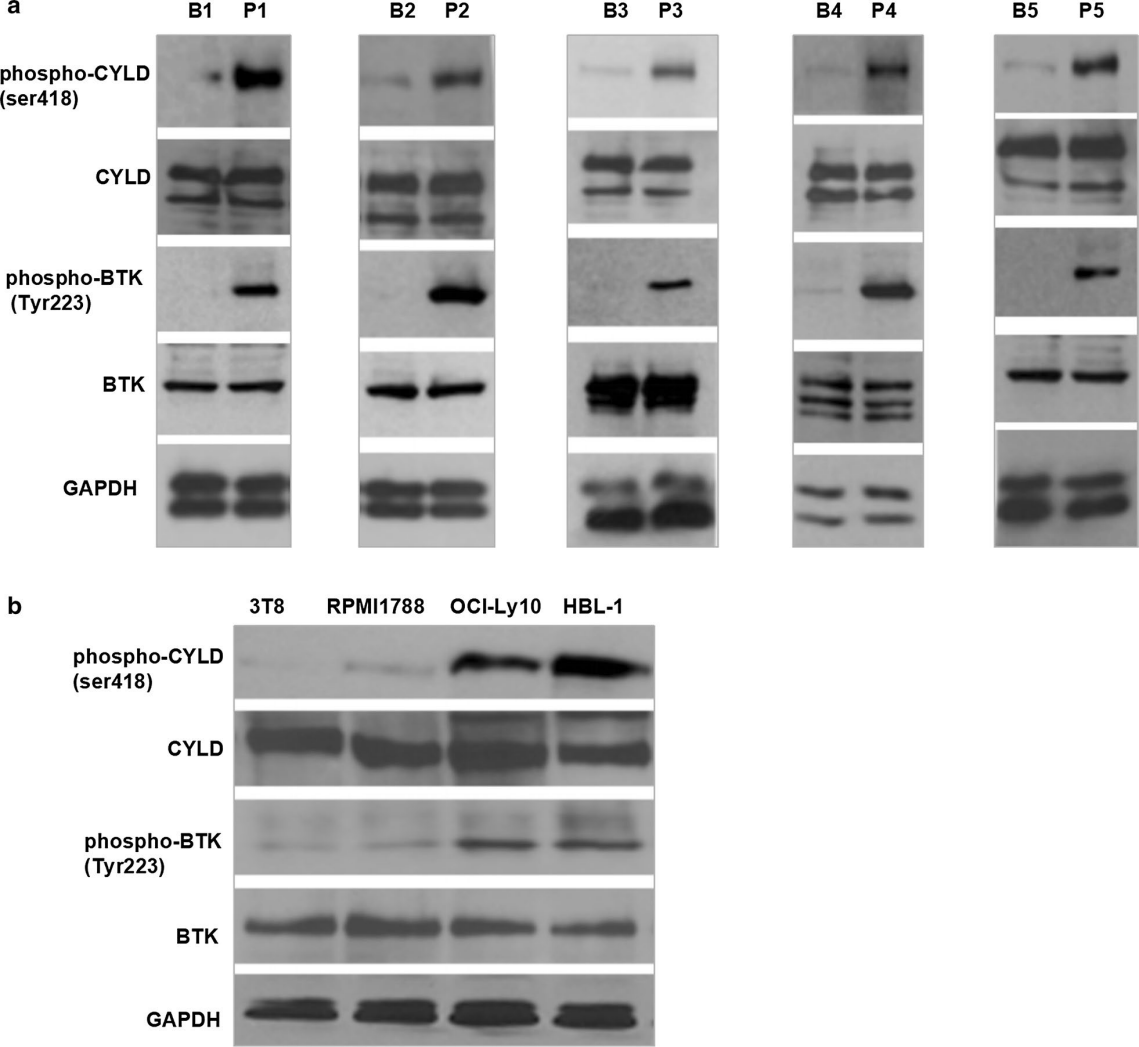
CYLD was highly phosphorylated along with BTK phosphorylated in non-GCB-DLBCL. **a** Protein was extracted from five non-GCB-DLBCL patients (P1–P5) intranodal samples, five lymphadenitis patients inflammatory hyperplasia lymph node samples (B1–B5) as control, blotted sequentially for phospho-CYLD (ser418)/CYLD, phospho-BTK (Tyr223)/BTK, and GAPDH as a loading control. **b** Protein was extracted from non-GCB-DLBCL cell lines OCI-Ly10 and HBL-1, the human peripheral blood B lymphocyte cell line RPMI 1788 and Jurkat T-cell subclone 3T8 as control, and blotting sequentially for phospho-CYLD (ser418)/CYLD, phospho-BTK (Tyr223)/BTK, and GAPDH as a loading control

Five tumor invasive lymph node samples from newly diagnosed non-GCB-DLBCL lymphomas (P1–P5) and five inflammatory hyperplasia lymph node samples from lymphadenitis patients (B1–B5) as control were collected. The clinical characters of these five non-GCB-DLBCL patients see Table 1. Non-GCB-DLBCL cell lines OCI-Ly10 and HBL-1 were detected. Lysates from Jurkat T cells (clone 3T8) and human peripheral blood B lymphocyte cell line RPMI1788 as the negative controls were also detected. Expression levels of phospho-CYLD (at serine 418)/total-CYLD (p-CYLD/CYLD) and phospho-BTK (at tyrosine 223)/total-BTK (p-BTK/BTK) were detected by Western Blotting. There was no significant difference in total CYLD or total BTK expression levels between non-GCB-DLBCL samples (non-GCB-DLBCL patients’ samples; OCI-Ly10 and HBL-1 cell lines) and control samples (lymphadenitis patients’ samples; 3T8 and RPMI1788 cell lines). But there were significant difference in phospho-BTK and phospho-CYLD in the non-GCB-DLBCL patient samples (P1–P5) compared with the lymphadenitis patient samples (B1–B5) (Fig. 1a). The same changes were also showed in non-GCB-DLBCL cell lines (OCI-Ly10 and HBL-1 cell lines) compared with the Jurkat T-cell 3T8 and the human peripheral blood B lymphocyte cell line RPMI 1788 (Fig. 1b). The synchronously change of CYLD phosphorylation and BTK phosphorylation suggested that CYLD phosphorylation should be regulated through inhibition of BTK phosphorylation, which might regulate tumor cell death in non-GCB-DLBCL.

**Table 1:** Clinical characteristics of five DLBCL patients

No.	Patient ID	COO IHC	Immunophenotype	FISH	IPI score^a^	Ann Arbor stage^b^
DLBCL1	D653661	Non-GCB	CD20 + , CD10 −, BCL6 + , MUM1 + , BCL2 +	IGH, BCL6 and MYC not rearranged, 3 copies of BCL6 noted indicating trisomy 3q, TP53 deleted	4	III
DLBCL2	D910596	Non-GCB	CD20 + , CD10 −, BCL6-, MUM1 + , BCL2 +	IGH, BCL6 and MYC not rearranged, one copy of IGH deleted, TP53 deleted	4	III
DLBCL3	D836558	Non-GCB	CD20 + , CD10 −, BCL6-, MUM1 + , BCL2 +	IGH and BCL6 rearranged, TP53 deleted	5	IV
DLBCL4	D982560	Non-GCB	CD20 + , CD10 −, BCL6 −, MUM1 −, BCL2-	IGH, BCL6 and MYC not rearranged, TP53 deleted	4	III
DLBCL5	D987446	Non-GCB	CD20 + , CD10 −, Bcl-6 + , MUM1 + , BCL2 +	IGH and BCL6 rearranged, BCL2 or MYC not rearrangment, TP53 deletion	3	III

^a^IPI score, International Prognostic Index score

^b^Ann Arbor stage according to Ann Arbor-Cotswald staging (1989)

### BTK inhibitors down-regulated CYLD phosphorylation in non-GCB-DLBCL

**Fig.2:**
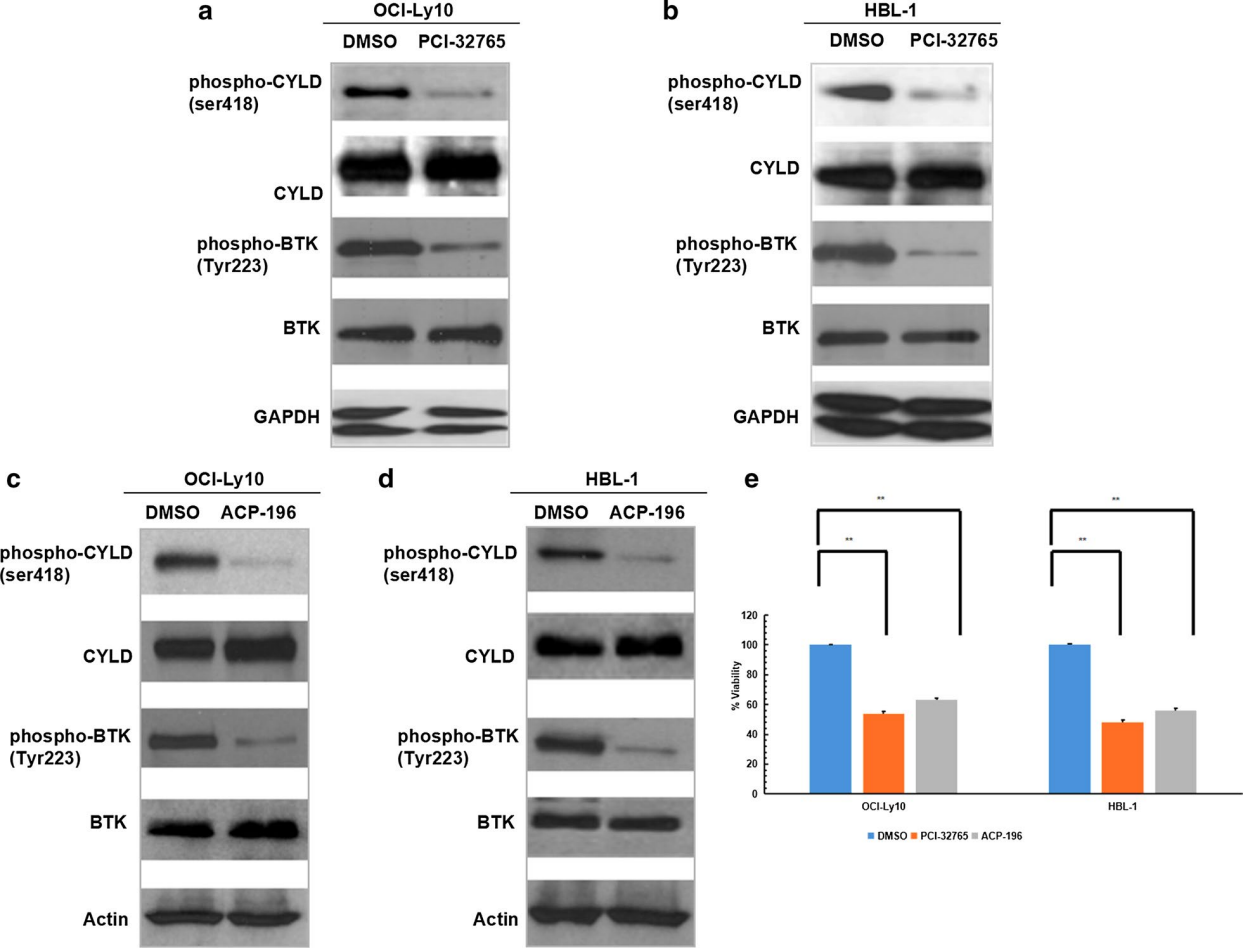
BTK inhibitors down-regulated CYLD phosphorylation along with BTK phosphorylation in non-GCB -DLBCL. **a–d** OCI-Ly10 and HBL-1 cells were treated with DMSO or 10 μM PCI-32765 (**a**, **b**) or 10 μM ACP-196 (**c**, **d**) for 48 h, lysates were sequentially blotted with the indicated antibodies. **e** OCI-Ly10 and HBL-1 cells were treated with DMSO or 10 μM PCI-32765 or 10 μM ACP-196 for 72 h, cell viability was assessed using CellTiter-Glo assay. The ATP level for each cell line treated with DMSO was set at 100%. The bars represent the mean ± SD from three independent experiments. **P < 0.01

OCI-Ly10 and HBL-1 cells were treated with BTK inhibitors (BTKis) ibrutinib (PCI-32765) and acalabrutinib (ACP-196), respectively, DMSO was used as control. Western blotting showed that BTKis inhibited the phosphorylation of both CYLD and BTK in these two cell lines (Fig. 2a–d). And ATP viability assay showed BTKis induced cell death in these two cell lines (Fig. 2e). These results further suggested that CYLD phosphorylation is an essential mediator in this BTKis downstream pathway to promote cell death in non-GCB-DLBCL.

### BTK inhibitors induced CYLD dependent apoptosis in non-GCB-DLBCL cell lines

**Fig.3:**
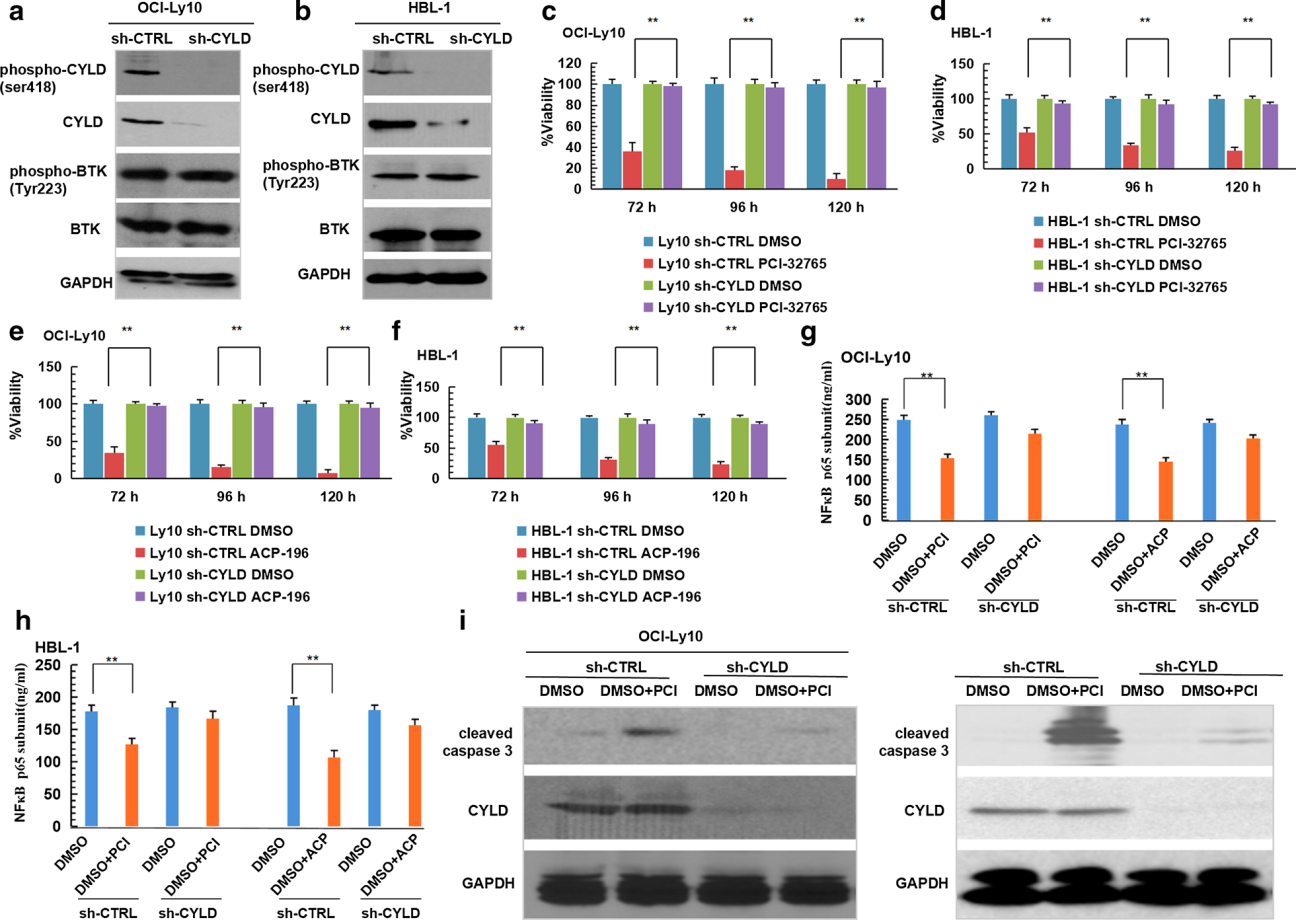
BTK inhibitors induced CYLD dependent apoptosis in non-GCB-DLBCL. **a, b** OCI-Ly10 (**a**) and HBL-1 (**b**) cells were transfected with non-targeting or CYLD-targeting shRNA. Lysates were blotted with the indicated antibodies. **c–f** Control or CYLD-deficient OCI-Ly10 and HBL-1 cells were treated with DMSO or 10 μM PCI-32765 (**c**, **d**) or 10 μM ACP-196 (**e**, **f**) for 72-120 h, cell viability was assessed using CellTiter-Glo assay. **g**, **h** Control or CYLD-deficient OCI-Ly10 and HBL-1 cells were treated with DMSO or 10 μM PCI-32765 (**g**) or 10 μM ACP-196 (**h**) for 72 h, NFκB p65 subunit expression levels were measured by ELISA. **i**,** j** Control or CYLD-deficient OCI-Ly10 (**i**) and HBL-1 (**j**) cells were treated with DMSO or 10 μM PCI-32765 for 48 h, lysates were blotted with the indicated antibodies. The ATP level for each cell line treated with DMSO at each time point was set at 100%. The bars represent the mean ± SD from three independent experiments. **P < 0.01

CYLD-knock-down OCI-Ly10 and HBL-1 cells (sh-CYLD) and control cells (sh-CTRL) were generated through CYLD-targeting shRNA or non-targeting short hairpin RNA transfection. Western Blotting showed CYLD had been knocked down but no impact on total BTK or BTK phosphorylation in the two cell lines (Fig. 3a, b). ATP viability assay showed that cell death was rescued in CYLD-knock-down cells treated with BTKis (PCI-32765 and ACP-196) compared with control cells (Fig. 3c–f), which suggested BTKis induced CYLD dependent cell death in non-GCB-DLBCL. Phosphorylated p65 sub-unit expression levels, which represented NFκB activation, were also measured. BTKis could inhibit NFκB activation, which suggested that cell death in non-GCB-DLBCL induced by BTKis might correlated with down-regulating NFκB signaling pathway (Fig. 3g, h). However, when CYLD was knocked down, BTKis could not inhibit NFκB activation, which suggested that BTKis down-regulated NFκB signaling pathway was CYLD dependent in non-GCB-DLBCL (Fig. 3g, h). Meanwhile, apoptosis-related protein caspase-3 cleavage products (cleavage caspase3) expression was detected. The expression of cleavage caspase3 under BTK inhibitor PCI-32765 treatment in CYLD-knock-down cells were lower than in control cells. Took together, the above experimental results suggested that BTKis induced CYLD dependent apoptosis in non-GCB-DLBCL cells, and the down-regulation of the NFκB signaling pathway activation may be a crucial mechanism (Fig. 3i, j).

### BTK inhibitor ibrutinib down-regulated CYLD phosphorylation and inhibited tumor growth in xeograft mice model

**Fig.4:**
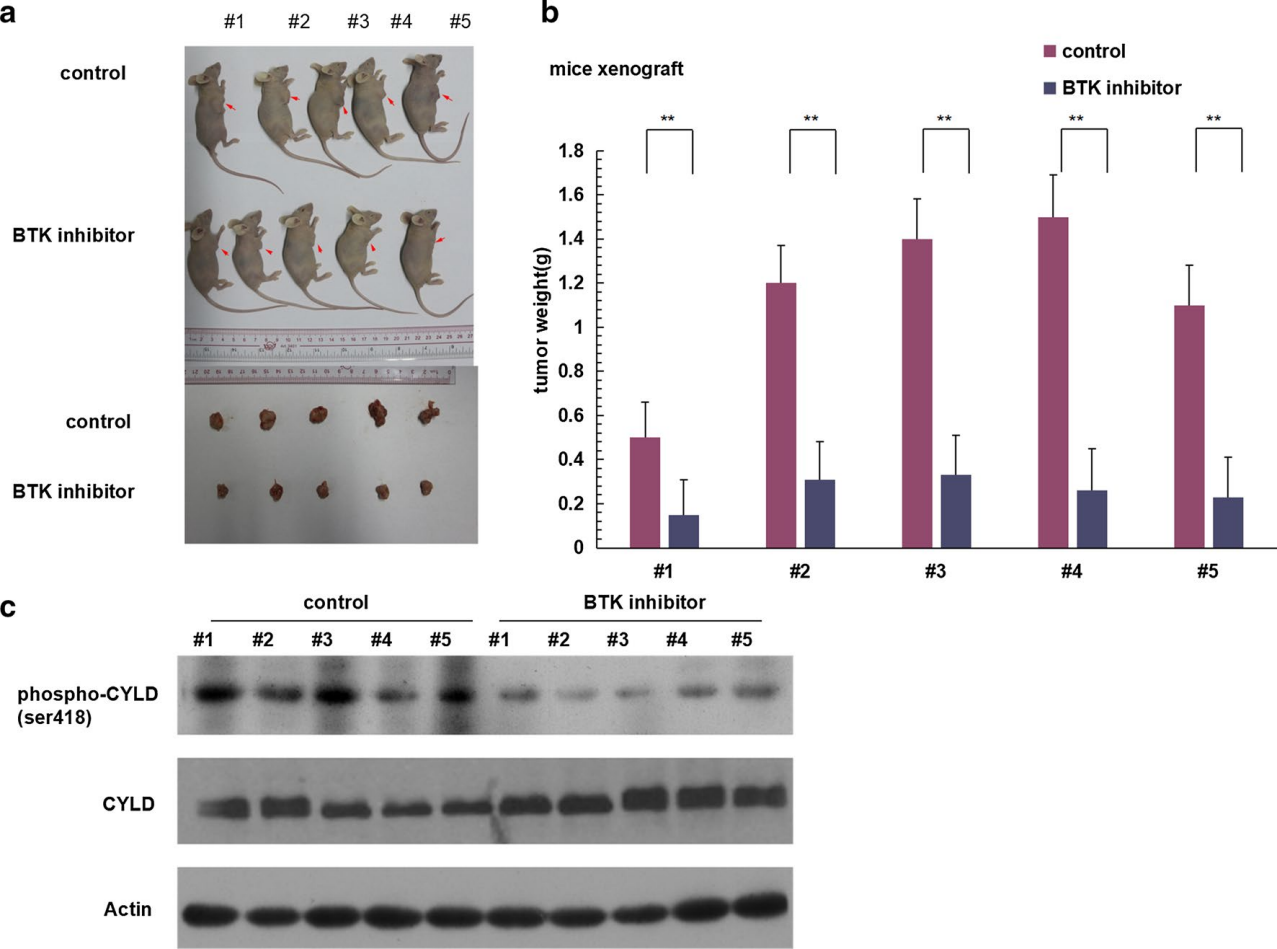
BTK inhibitors down-regulated CYLD phosphorylation and inhibited tumor growth in vivo. **a**, **b** CB17-SCID mice were injected subcostally with 5.0 × 10^6^ OCI-ly10 cells or placebo control, tumor growth (**a**) and tumor volume (**b**) were assessed. The bars represent the mean ± S.D. from three independent experiments. **P < 0.01. **c** Non-GCB-DLBCL xenografted mice were treated with placebo or ibrutinib 12 mg/kg, tumor tissue lysates were blotted with the indicated antibodies to phospho-CYLD/CYLD and GAPDH

Non-GCB-DLBCL xeograft mice models were constructed by inoculating OCI-Ly10 cells.The tumor volume of mice were significantly smaller in BTKis ibrutinib treatment group compared with placebo-treated group on day 18 (P = 0.029) (Fig. 4a, b). Proteins were extracted from the tumor tissue and Western blotting showed that the expression level of CYLD phosphorylation was significantly lower in BTKis group compared with in control group (Fig. 4c). These results suggested that BTKis inhibited tumor development in non-GCB-DLBCL mice model by down-regulating CYLD phosphorylation,which further confirmed that CYLD phosphorylation is an essential mediator in BTKis downstream pathway to promote tumor cell death in non-GCB-DLBCL in vivo.

### BTK inhibitors can enhance the apoptosis of non-GCB-DLBCL cells induced by rituximab through down-regulating the phosphorylation of CYLD

**Fig.5:**
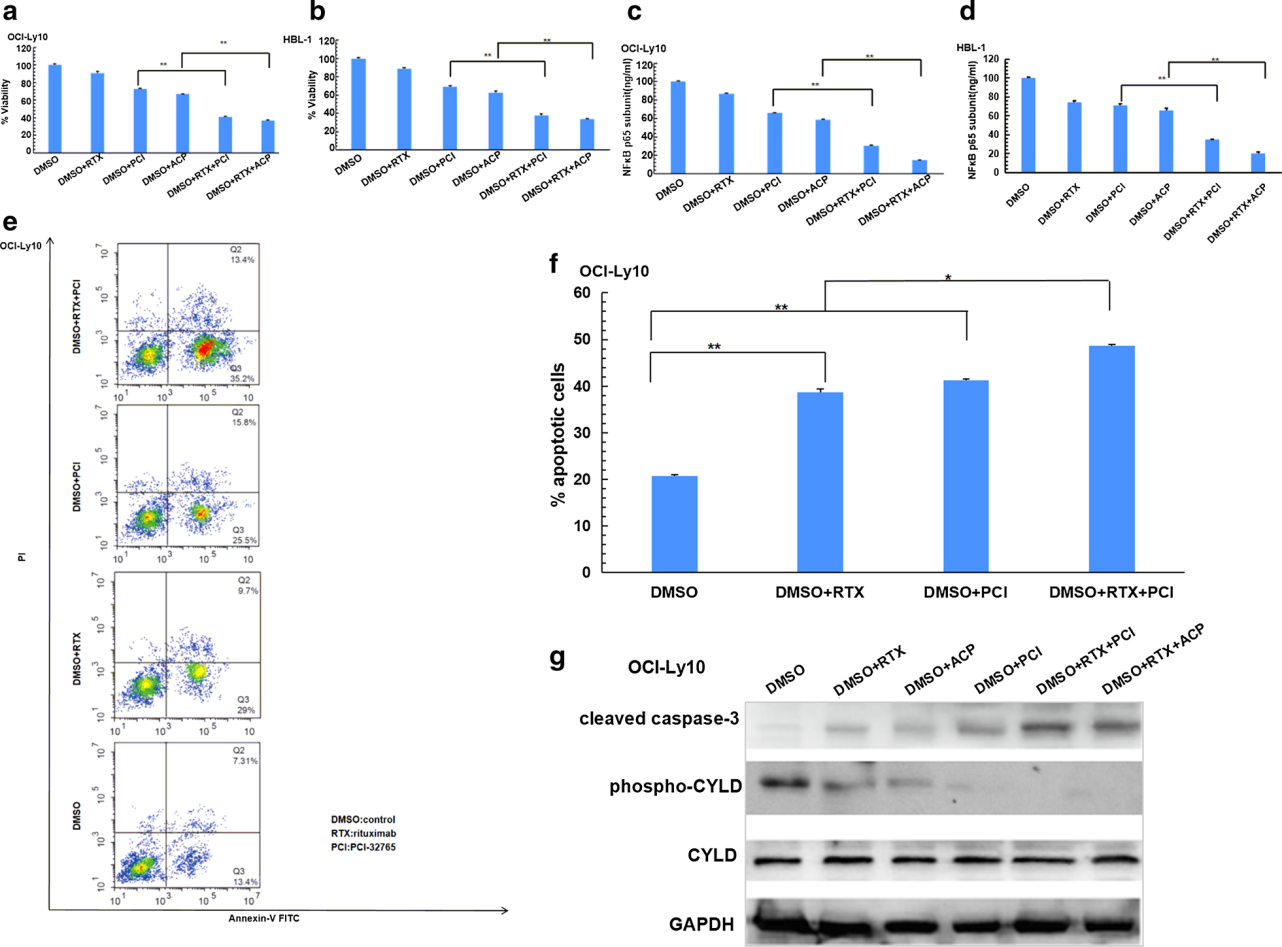
BTK inhibitors reduced CYLD phosphorylation and enhanced apoptosis induced by rituximab of non-GCB-DLBC. **a**, **b** OCI-Ly10 (**a**) and HBL-1 (**b**) cells were treated with DMSO, 50 μg/ml rituximab, 10 μM PCI-32765, 10 μM ACP-196, combination of 50 μg/ml rituximab and 10 μM PCI-32765, combination of 50 μg/ml rituximab and 10 μM ACP-196 for 72 h, cell viability was detected by CellTiter-Glo assay. **c**, **d** OCI-Ly10 (**c**) and HBL-1 (**d**) cells were treated with the same conditions as **a** and **b**, NFκB p65 subunit activation was measured by ELISA. **e** OCI-Ly10 cells were treated with 50 μg/ml rituximab, 10 μM. PCI-32765, 50 μg/ml rituximab + 10 μM PCI-32765 for 48 h, respectively. The apoptotic cell population including early and late apoptosis cells was quantified using an ApoDETECT Annexin V-FITC Kit and analyzed by flow cytometry. **f** Bars indicate the frequency of apoptotic OCI-Ly10 cell population including early and late apoptosis cells. **g** OCI-Ly10 cells were treated with 10 μM PCI-32765 or 10 μM ACP-196, with or without 50 μg/ml rituximab for 48 h, lysates were blotted with the indicated antibodies. The ATP level for each cell line treated with DMSO was set at 100%. The bars represent the mean ± SD from three independent experiments. *P < 0.05, **P < 0.01

OCI-Ly10 and HBL-1 cells were treated with BTKis ibrutinib (PCI-32765) or acalabrutinib (ACP-196), respectively, with or without CD20 monoclonal antibody rituximab. CYLD phosphorylation and apoptosis of non-GCB-DLBCL cells were detected. ATP viability assay results showed that BTK inhibitors can enhance the cell death of non-GCB-DLBCL cells induced by rituximab (Fig. 5a, b). NFκB p65 sub-unit were detected by ELISA and the results showed that BTK inhibitors can enhance the inactivation of NFκB induced by rituximab (Fig. 5c, d). OCI-Ly10 cells were treated with BTK ibrutinib (PCI-32765), with or without rituximab. Apoptosis cells population were detected by the flow cytometry. The results indicated that rituximab induced more apoptosis of non-GCB-DLBCL cells than DMSO, and BTK inhibitor could enhance the apoptosis of non-GCB-DLBCL cells induced by rituximab (Fig. 5e, f). CYLD phosphorylation and cleavage caspase3 were detected by Western blotting, and the results further indicated that BTK inhibitors could enhance the apoptosis of non-GCB-DLBCL cells induced by rituximab through down-regulating the phosphorylation of CYLD (Fig. 5g).

### BTK inhibitor PCI-32765 promoted CYLD-dependent apoptosis in rituximab-resistant non-GCB-DLBCL cells

**Fig.6:**
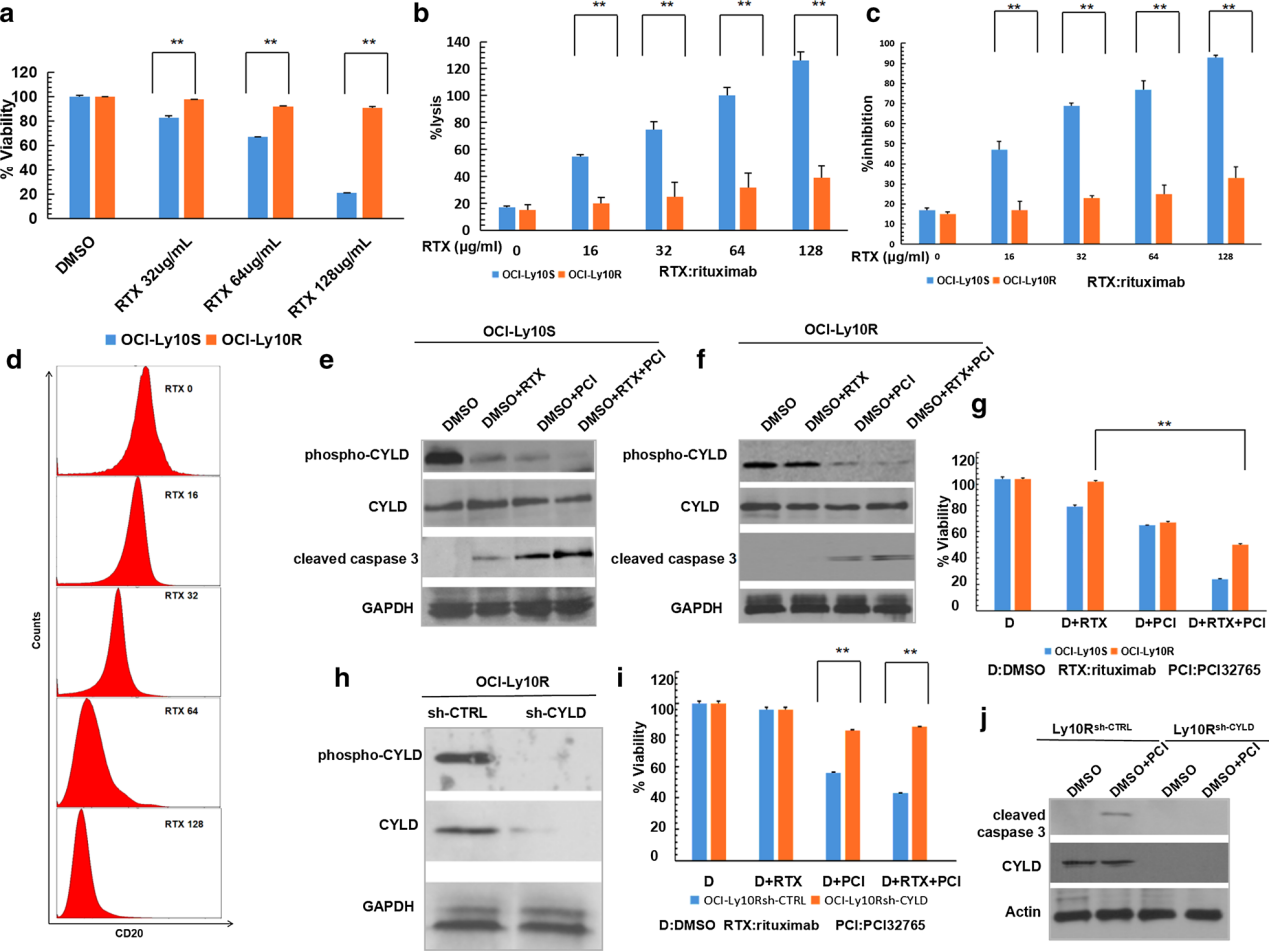
BTK inhibitors promoted CYLD-dependent apoptosis of rituximab-resistant non-GCB-DLBCL cells. **a** Construction of rituximab-resistant cell line by the concentration gradient method. Rituximab-resistant OCI-Ly10 cells (OCI-Ly10R) and rituximab-sensitive OCI-Ly10 cells (OCI-Ly10S) were treated with DMSO or rituximab and cell viability was assayed by CellTiter-Glo assay. **b** OCI-Ly10R and OCI-Ly10S cells were labeled of 51Cr, lysis was collected individually and γ emission was measured. **c** OCI-Ly10R and OCI-Ly10S cells were treated with CCK-8 reagent, the OD value at 450 nm was measured and the inhibition rate was calculated. **d** Parental OCI-Ly10 cells were exposed to sequentially increasing concentrations of rituximab for 48 h. Cells then stained with anti-CD20-FITC antibody and phenotyping of OCI-Ly10S and OCI-Ly10R cells were performed on flow cytometer. **e**, **f** OCI-Ly10S (**e**) and OCI-Ly10R (**f**) cells were treated with DMSO, 50 μg/ml rituximab, 10 μM PCI-32765 or combination of 50 μg/ml rituximab and 10 μM PCI-32765 for 48 h. Lysates were blotted with the indicated antibodies. **g** OCI-Ly10S and OCI-Ly10R cells were treated with DMSO, 50 μg/ml rituximab, 10 μM PCI-32765 or combination of 50 μg/ml rituximab and 10 μM PCI-32765 for 72 h. Cell viability was assayed by CellTiter-Glo assay. **h** OCI-Ly10R cells were transduced with lentiviruses encoding a non-targeting or CYLD-targeting shRNA. Lysates were blotted with the indicated antibodies. **i** Control or CYLD-deficient OCI-Ly10R cells were treated with DMSO or 50 μg/ml rituximab or 10 μM PCI-32765 or combination of 50 μg/ml rituximab and 10 μM PCI-32765 for 72 h. Cell viability was assayed by CellTiter-Glo assay. **j** Control or CYLD-deficient OCI-Ly10R cells were treated with DMSO or 10 μM PCI-32765 for 48 h. Lysates were blotted with the indicated antibodies. The ATP levels for each cell line treated with DMSO were set at 100%. The bars represent the mean ± SD from three independent experiments. **P < 0.01

Rituximab-resistant (OCI-Ly10R) or -sensitive(OCI-Ly10S) OCI-Ly10 cells were generated and ATP viability assay was done to show the OCI-Ly10S cells but not the OCI-Ly10R cells died under rituximab (32, 64, 128 μg/ml) treatment (Fig. 6a). Standard ^51^Cr release assays and standard Cell Counting Kit-8 (CCK-8) assay were done to assess rituximab-mediated CDC (Fig. 6b, c). The flow cytometry study using an Alexa conjugated rituximab showed that CD20 expression of OCI-Ly10 cells decreased corresponding to the increasing rituximab concentrations (RTX 16, 32, 64, 128 μg/ml) (Fig. 6d). The above results indicated that the rituximab-resistant cells were successfully generated. OCI-Ly10R and OCI-Ly10S cells were treated by BTK inhibitor PCI-32765 (10 μM) and rituximab (50 μg/ml). Western blotting showed that in OCI-Ly10S cells, BTK inhibitor PCI-32765 could enhance the apoptosis of non-GCB-DLBCL cells induced by rituximab through down-regulating the phosphorylation of CYLD (Fig. 6e). But in OCI-Ly10R cells, BTK inhibitor PCI-32765 but not rituximab could reduce CYLD phosphorylation and induce apoptosis (Fig. 6f). The further ATP viability assay also confirmed that BTK inhibitor PCI-32765 could induce cell death even when the cells were resistant to rituximab (Fig. 6g). Sh-CYLD or sh-CTRL plasmids transfection were done in OCI-Ly10R cells to generate CYLD-knock-down OCI-Ly10R cells(OCI-Ly10R^sh-CYLD^) and control OCI-Ly10R cells(OCI-Ly10R^sh-CTRL^) (Fig. 6h). ATP viability assay showed that knocking down CYLD in OCI-Ly10R cells could rescue the cell death induced by BTK inhibitor PCI-32765 (Fig. 6i). And the Western blotting further confirmed that BTK inhibitor PCI-32765 could not induce apoptosis in CYLD-knock-down OCI-Ly10R cells (Fig. 6j). To sum up, these results indicated that BTK inhibitor promoted CYLD-dependent apoptosis in rituximab-resistant non-GCB-DLBCL cells.

## Discussion

In this study, we put forward a hypothesis of BTK inhibitors (BTKis) could regulate CYLD phosphorylation to promote apoptosis through down-regulating NFκB signaling pathway in non-GCB-DLBCL and we did experiments in vivo and in vitro to demonstrate this hypothesis. Our results indicated that CYLD phosphorylation were significant different between non-GCB-DLBCL samples and control samples. We demonstrated that BTKis could down-regulate CYLD phosphorylation and induce CYLD dependent apoptosis in non-GCB-DLBCL via inactivating NFκB pathway. And we further demonstrated that this apoptosis induced by BTKis in non-GCB-DLBCL was CYLD dependent. Our results indicated that CYLD phosphorylation is a potential target for pharmacologic modification to improve therapeutic outcomes in non-GCB-DLBCL.

BTK inhibitors (BTKis) are new drugs in B cell lymphoma, which have been used in newly diagnosed relapse/refractory (R/R) or rituximab-resistant DLBCL [36]. BTK is expressed in B cells, and plays a critical role in B-cell receptor (BCR) signaling pathway, which regulates cell survival [37–39]. Previous researches had showed high expression level of BTK phosphorylation in B cell lymphoma, which represented BTK activation and had correlation with lymphoma development [40]. BTKis inhibited BTK phosphorylation will inhibit lymphoma cells proliferation and increase overall survival in preclinical Burkitt lymphoma, and this effect could be enhanced by rituximab [41]. It is well known that constitutive activation of NFκB pathway is known as a hallmark of non-GCB-DLBCL, inhibition of NFκB induces apoptosis in different DLBCL cell lines, specifically in non-GCB-DLBCL [42, 43]. Therefore, besides promoting tumor cells proliferation, BTK activation also can inhibit apoptosis through NFκB signaling pathway in non-GCB-DLBCL [17]. But what are the regulatory points of downstream signaling pathway mediated by BTK phosphorylation inhibition are still unclear.

CYLD is considered to be a tumor suppressor widely existing in a variety of tumors [44–46]. However, the protein expression levels of CYLD were not consistent in different cancer species. In breast cancer, CYLD protein expression levels were lower in tumor than in normal tissues [47]. But in early-stage colorectal cancer CYLD protein expression levels were high and gradually decline with disease progression [48]. There are few studies on the protein expression levels of CYLD in hematological malignances. In lymphoma like CLL, CYLD protein expression levels was lower in tumor invasive lymph node than in normal lymph node samples [49]. But in our previous research about T cell lymphoma,total CYLD protein expression levels was no significant difference between tumor invasive lymph node and normal lymph node samples [24]. These findings suggest that there are different mechanisms of functional regulation of CYLD in different tumors. The expression levels of CYLD protein and it underlying mechanisms in non-GCB-DLBCL are still explored. Phosphorylation is an important process down-regulating CYLD activity [22, 23]. However, most of the previous studies on CYLD regulating the occurrence and development of hematological malignances focused on the CYLD gene mutation or CYLD protein cleavage [50–54]. The research on how CYLD phosphorylation regulate the occurrence and development of hematological malignances is still lacking. Our previous study indicated high protein expression levels of CYLD phosphorylation in adult T-cell leukemia/lymphoma (ATLL) and regulating CYLD phosphorylation could regulate CYLD ubiquitin activity to improve ATLL cells apoptosis [24]. Whether CYLD phosphorylation is also a regulator for non-GCB-DLBCL apoptosis needs further research. CYLD is a negative regulator of NFκB which suppress many kind of tumors through its deubiquitination enzyme function [20]. Previous researches in CLL indicated that BTK inhibitor ibrutinib could largely increase CYLD activity through increasing CYLD miRNA transcription, which could inhibit cells proliferation in CLL [25]. But whether BTKis can regulate CYLD phosphorylation to induce apoptosis via down-regulating NFκB in non-GCB-DLBCL remains unclear. Our study indicated that there was no significant change in NFκB activity in both lymphoma cell lines with CYLD knockout (OCI-Ly10 and HBL-1) after BTK inhibitor treatment, suggesting that BTKis down-regulated NFκB activity is dependent on the CYLD pathway.

More and more clinical researches showed that many relapsed/refractory non-GCB-DLBCL (R/R non-GCB-DLBCL) cases were resistant to CD20 monoclonal antibody rituximab [4, 5]. Mechanisms of resistance have been identified as below: altered CD20 antigen expression or binding, impacted on complement dependent cytoxicity (CDC) or antibody-dependent cellular cytotoxicity (ADCC) effects, altered intracellular signaling, and inhibition of direct cell death induction [55, 56]. In recent years, a profound understanding of the tumor micro-environment and targeting the apoptotic pathway has led to promising breakthroughs. We know that resistance may be driven by unique patient-, disease-, and antibody-related factors. Understanding the mechanisms of resistance to rituximab will develop new strategies to overcome resistance and re-sensitize cancer cells to these biological agents [56]. We developed experiments and successfully demonstrated BTKis could still promote apoptosis through regulating CYLD phosphorylation in rituximab resistant non-GCB-DLBCL. In addition, we found that knocking down CYLD in rituximab-resistant non-GCB-DLBCL cells would attenuate this BTK inhibitors induced apoptosis, consequently, further confirmed that this BTK inhibitors induced CYLD dependent apoptosis in rituximab-resistant non-GCB-DLBCL. Our results indicated that CYLD phosphorylation is a potential pharmacologic target which can sensitize rituximab-resistant non-GCB-DLBCL to BTK inhibitors. Our findings provide experimental basis for new strategies to reverse rituximab resistance. It is hopeful to improve clinical outcomes in R/R non-GCB-DLBCLs which are resistant to rituximab.

In the future, we will further investigate the deubiquitinase function of CYLD in non-GCB-DLBCL and in different types of lymphoma. Resistance to ibrutinib has been found in clinical practice. Although experimental studies have shown new BTK inhibitor ARQ531 is effective for ibrutinib resistant patients, clinical application of this compound is still a long way off [57]. Meanwhile, not only BTKis but also more and more new drugs emerge and acquire good clinical outcomes in non-GCB-DLBCL such as Lenalidomide [58–61]. Since previous basic research indicated that CYLD is a key adaptor that regulates various signaling pathways to modulate diverse physiological processes including immune responses [62], we also want to further investigate the potential effects of Lenalidomide regulating CYLD phosphorylation in non-GCB-DLBCL.

## Conclusion

In this study, we demonstrated that CYLD phosphorylation is a potential regulator for non-GCB-DLBCL development in vitro and in vivo. These results provide an experimental basis and a new strategy for improving the clinical therapeutic effect in patients with non-GCB-DLBCL, especially patients resistant to rituximab.

## Data Availability

The datasets used and analyzed during the current study are available from the corresponding author on reasonable request. This research was approved by the Ethics Committee of Guangzhou First People’s Hospital (K-2017-105-01). Written informed consent was obtained from all participants. All animal experimental procedures and protocols were approved by the Institutional Animal Care and Use Committee at Guangzhou First People’s Hospital.

## Abbreviations

DLBCL: Diffuse large B-cell lymphoma
NHL: Non-Hodgkin’s lymphoma
GCB-DLBCL: Germinal center B-cell-like DLBCL
non-GCB-DLBCL: Non-germinal center B-cell-like DLBCL
CD20 mAb: CD20 monoclonal antibody
RTX: Rituximab
CHOP: Cyclophosphamide, vincristine, adriamycin, and prednisone
R/R non-GCB-DLBCL: Relapsed/refractory non-GCB-DLBCL
BTK: Bruton’s tyrosine kinase
CLL/SLL: Chronic lymphocytic leukemia/small lymphocytic lymphoma
MCL: Mantle cell lymphoma
NCCN: National Comprehensive Cancer Network
BCR: B-cell receptor
NFκB: Nuclear transcription factor κB
OCT: Optimum cutting temperature compound
CDC: Complement-dependent cytotoxicity
ADCC: Antibody-dependent cellular cytotoxicity
